# Two-dimensional biphenylene: a promising anchoring material for lithium-sulfur batteries

**DOI:** 10.1038/s41598-022-08478-5

**Published:** 2022-03-17

**Authors:** Hiba Khaled Al-Jayyousi, Muhammad Sajjad, Kin Liao, Nirpendra Singh

**Affiliations:** 1 Department of Mechanical Engineering, Khalifa University of Science and Technology, 127788 Abu Dhabi, United Arab Emirates; 2 Department of Physics, Khalifa University of Science and Technology, 127788 Abu Dhabi, United Arab Emirates; 3 Department of Aerospace Engineering, Khalifa University of Science and Technology, 127788 Abu Dhabi, United Arab Emirates

**Keywords:** Materials science, Materials for energy and catalysis, Batteries

## Abstract

Trapping lithium polysulfides (LiPSs) on a material effectively suppresses the shuttle effect and enhances the cycling stability of Li–S batteries. For the first time, we advocate a recently synthesized two-dimensional material, biphenylene, as an anchoring material for the lithium-sulfur battery. The density functional theory calculations show that LiPSs bind with pristine biphenylene insubstantially with binding energy ranging from −0.21 eV to −1.22 eV. However, defect engineering through a single C atom vacancy significantly improves the binding strength (binding energy in the range −1.07 to −4.11 eV). The Bader analysis reveals that LiPSs and S8 clusters donate the charge (ranging from −0.05 e to −1.12 e) to the biphenylene sheet. The binding energy of LiPSs with electrolytes is smaller than those with the defective biphenylene sheet, which provides its potential as an anchoring material. Compared with other reported two-dimensional materials such as graphene, MXenes, and phosphorene, the biphenylene sheet exhibits higher binding energies with the polysulfides. Our study deepens the fundamental understanding and shows that the biphenylene sheet is an excellent anchoring material for lithium-sulfur batteries for suppressing the shuttle effect because of its superior conductivity, porosity, and strong anchoring ability.

## Introduction

Over the last three decades, lithium-ion rechargeable batteries have gained vast popularity due to their low self-discharge, ample energy storage, stable cycling performance, higher theoretical capacity, and specific energy density, which directly affected the development of energy storage technologies^1–3^. Li-ion batteries are environmental-friendly and prominent for portable electronics, as they offer much higher energy density than other rechargeable systems^4–6^. Furthermore, lithium-sulfur batteries are excellent for next-generation rechargeable batteries with a theoretical capacity of 1675 mAh g^−1^. and specific energy of 2600 Wh Kg^−1^, respectively, higher than commercial Li-ion batteries. In addition, the sulfur cathode has many merits of abundant resources, cheap and non-pollution^2,7,8^. Further advances in rechargeable batteries are essential to fulfill electric vehicles and energy storage demand.

Current research efforts are focused on overcoming the challenges that limit the efficiency of lithium-sulfur (Li–S) battery, such as capacity fade, the solubility of active species, discharge/charge characteristics, and the most notorious and intractable shuttle effect^9–11^. The shuttle effect is the migration of sulfur back and forth between the positive and negative electrodes in the charging and discharging process^12,13^. The S atoms also react with the lithium anode and form lithium polysulfides (LiPSs) Li_2_S_x_, 2 < *x* ≤ 8, which leads to corrosion, resulting in fast capacity fading and low Coulombic efficiency^14^. There are two main methods to suppress the shuttle effect, one is by preventing the diffusion of polysulfides into electrolytes, and the other is by using an anchoring material to block the migration of the polysulfides to the anode^15^. In the first case, polysulfides are diverted towards a material present in the cathode or electrolyte to decrease the solubility^16^. The second strategy is carried out by introducing a material to block the diffusion pathway of polysulfides. The diffusion process will be restrained when the binding of LiPSs clusters to the host material is more robust than that to the electrolytes.

A suitable anchoring material should have excellent conductivity, high surface area, porous structure, and high binding energy with the polysulfides^17,18^, preventing polysulfides from dissolving into electrolytes. Several two-dimensional (2D) materials have been proposed as anchoring materials employed to suppress the shuttling effect, such as graphene, OH, F, and S terminated Ti_2_C MXene^17^, porous vanadium nitride nanoribbon with graphene^19,20^, and boro-phosphorene^21^. The biphenylene (BPN), a planar fully *sp*^2^-hybridized allotrope of carbon, is recently synthesized^22^ where carbon atoms in BPN are arranged in square, hexagonal, and octagonal rings. It is metallic with a Dirac cone above the Fermi level, suggesting high carrier mobility and good conductivity^23^. Also, it shows a planar lattice which is one key factor for the design of batteries with a fast charge/discharge rate, so it is expected to exhibit improved performance as a potential anchoring material^24^. Therefore, the first-principles calculations are carried out to evaluate the potential of 2D BPN sheet as an anchoring material for Li–S batteries.

## Computational methods

The first-principles spin-polarized calculations are performed using the Vienna *ab-initio* Simulation Package (VASP)^25^. The projector augmented wave method is adopted to describe the ion–electron interactions^25^, which is treated by the generalized gradient approximation with the Perdew-Burke-Ernzerhof formalism^26^. The van der Waals interactions are implemented considering the scheme of Grimme (DFT-D3) with zero damping correction^27^. The plane-wave cutoff kinetic energy is set to 550 eV. The total ground state energy and force convergence criteria is set to 10^–6^ eV and 0.05 eV/Å, respectively. The interactions between two adjacent periodic images are eliminated by introducing a vacuum of 24 Å perpendicular to the sheet. A 3 × 3 × 1 supercell of BPN with the Monkhorst–Pack *k*-point mesh of 3 × 3 × 1 is used for geometry optimization and self-consistent calculations. The binding energy (*E*_*b*_) of LiPSs and S_8_ clusters with the BPN sheet is calculated using *E*_*b*_ = E_total_ − (E_biphenylene_ + E_LiPSs/S8_), where E_total_, E_biphenylene_, and E_LiPSs/S8_ refer to the total energy of biphenylene adsorbed with Li_2_S_x_/S_8_, pristine biphenylene, and the isolated Li_2_S_x_/S_8_ clusters, respectively.

## Results and discussion

**Figure 1:**
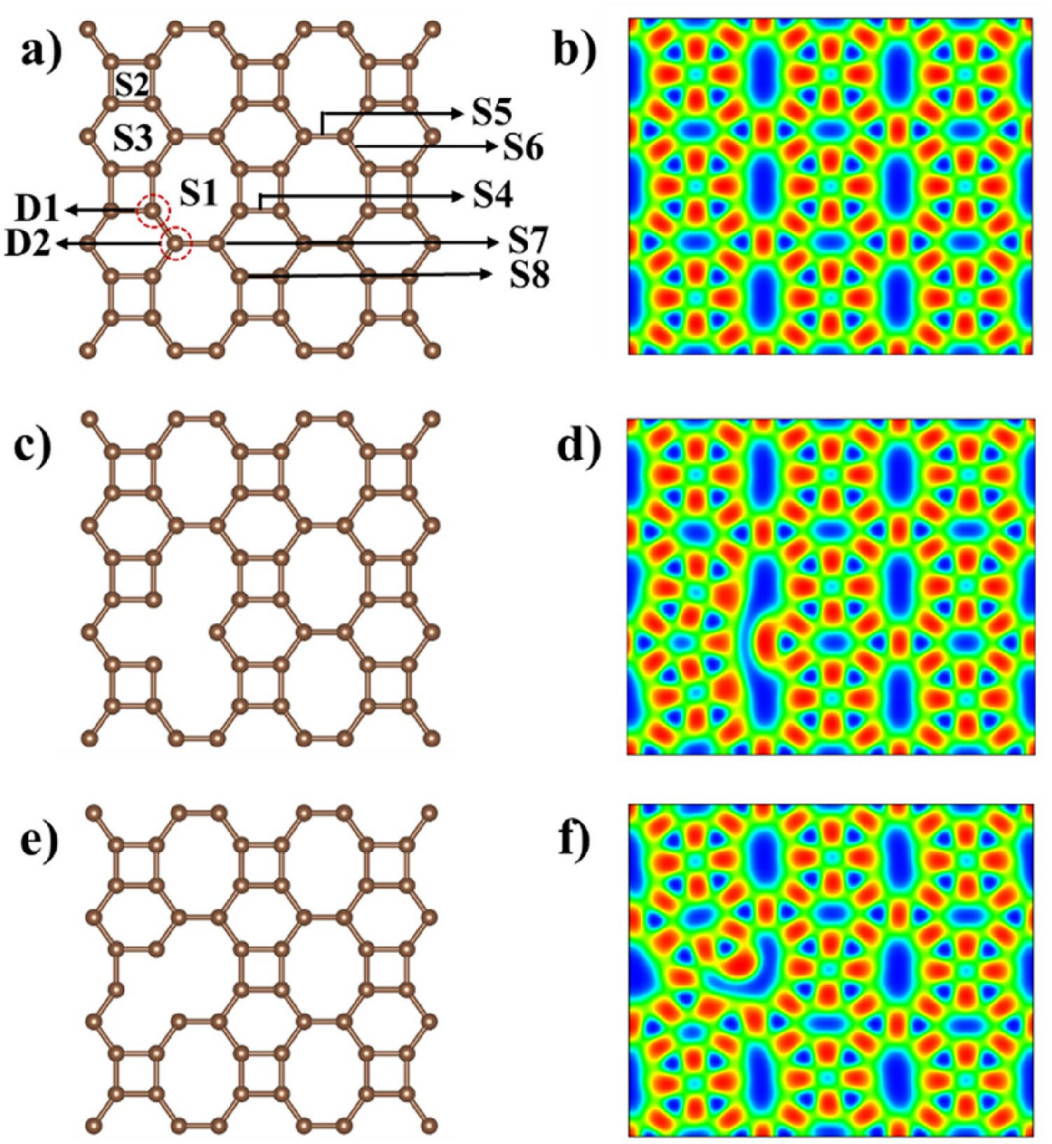
(**a**) Crystal structure of pristine BPN sheet with the possible adsorption sites (S1-S8) (**b**) The electron localization function of pristine BPN sheet (**c**,**e**) crystal structure of defective BPN sheet, and (**d**,**f**) the corresponding electron localization function.

The BPN sheet possesses a carbon lattice with a rectangular primitive unit cell formed by six C atoms having tetragon, hexagon, and octagon rings. The optimized lattice constants of the BPN sheet are 4.51 Å and 3.76 Å, consistent with the previous study^24^. The electron localization function along the (0 0 1) plane shows the underlying characteristics of C–C bonds (see Fig. 1b) and confirms that a large proportion of bonds in the BPN sheet are strong covalent bonds. In Fig. 1b, the red color shows intense localization and, in turn, a stronger bond. As illustrated in Fig. 1a, there are eight different adsorption sites. The S1, S2, and S3 sites are the octagon, tetragon, and hexagon hollows, respectively, and the S4, S5, and S6 sites are the distinctive bridges between the carbon atoms, and S7, S8 which are randomly chosen carbon atoms. Since lattice defects are inevitable during the synthesis process, the bindings of LiPSs and S_8_ are also investigated on the defective BPN sheet having a single C vacancy. Such defects can also be generated using a laser/electron beam of an appropriate energy bombardment^28^. There are two distinctive carbon atoms in the BPN sheet (marked with two dotted circles in Fig. 1a and named them D1 and D2, respectively), leading to two distinctive single vacancy defects. The structures with the single defect are illustrated in Fig. 1c–f combined with their respective electron localization function plots. In both cases, intense red color is noticed at the vacancy site, indicating a free electron (dangling bond), which directly affects the bonding of polysulfides with the BPN sheet. In addition, the defective BPN sheets exhibit a magnetic moment of a value of 1 μ_B_ in both D1 and D2 cases, which is directly reflected in the calculated density of states. Compared to pristine BPN, the defective BPN sheets DOS is asymmetrical, which can be explained by the fact that when a vacancy has created the relaxation of its nearest neighbor atoms and resulting charge redistribution saturate the orbitals of two of the three C atoms, and the remaining unsaturated carbon atom is responsible for the magnetic moment. The computed density of states of pristine BPN and defective sheets are available in the supplementary information (SI) (see Fig. S2). Figure S2 shows that the biphenylene sheet is metallic, resulting in better anchoring material than phosphorene. Much higher performance as an anchoring material is expected of its porous nature compared to graphene and MXene.

**Figure 2:**
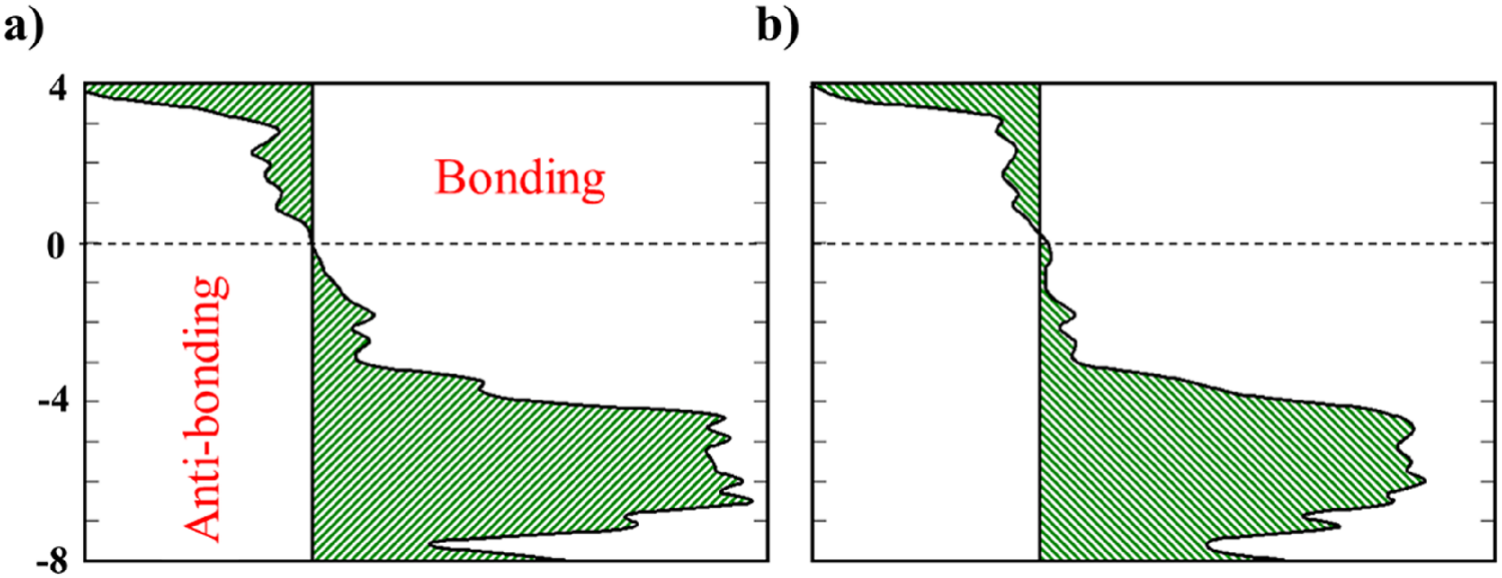
COHP for defected BPN sheet (**a**) corresponds to D1 defected sheet (**b**) corresponds to D2 defected sheet. The Fermi level is at zero.

The strength of the chemical bond in the defective BPN sheet is investigated with the aid of the projected crystal-orbital Hamilton population (-pCOHP) method, in which -pCOHP is divided into bonding and antibonding states, as shown in Fig. 2. In both cases, minor antibonding states near the Fermi level are observed, related to the electronic structure distortion resulting from the lone electron pair, making the two spin sublattices inequivalent. Apart from the minor antibonding, the C–C bonds contribute to the chemical stability of the bonding region. All the filled bands below the Fermi level contribute to chemical stability.

As cathode discharge, polysulfides significantly influence the capacity and cycling stability of Li–S batteries. The optimized most stable molecular structure of Li_2_S_x_ (*x* = 1, 2, 4, 6, 8) and S_8_ clusters are shown in the supplementary information (Fig. S3 in SI). The Li–S bond is more extended than the S–S bond, indicating that as the number of sulfur atoms increases, the high-order LiPSs can easily be ionized as Li^+^ and polysulfide anions, resulting in the so-called shuttle effect^9^. The binding energy of the LiPSs and S_8_ clusters with the BPN sheet is calculated by considering the most stable adsorption configuration (Fig. S1 in SI). Among the eight different adsorption sites, in most cases, LiPSs preferred to adsorb near the C–C bond of the octagon ring (S6 site in Fig. 1) because of charge accumulation. The maximum binding energy is −1.22 eV for Li_2_S, whereas the minimum binding energy of −0.43 eV is obtained for Li_2_S_4_.

**Figure 3:**
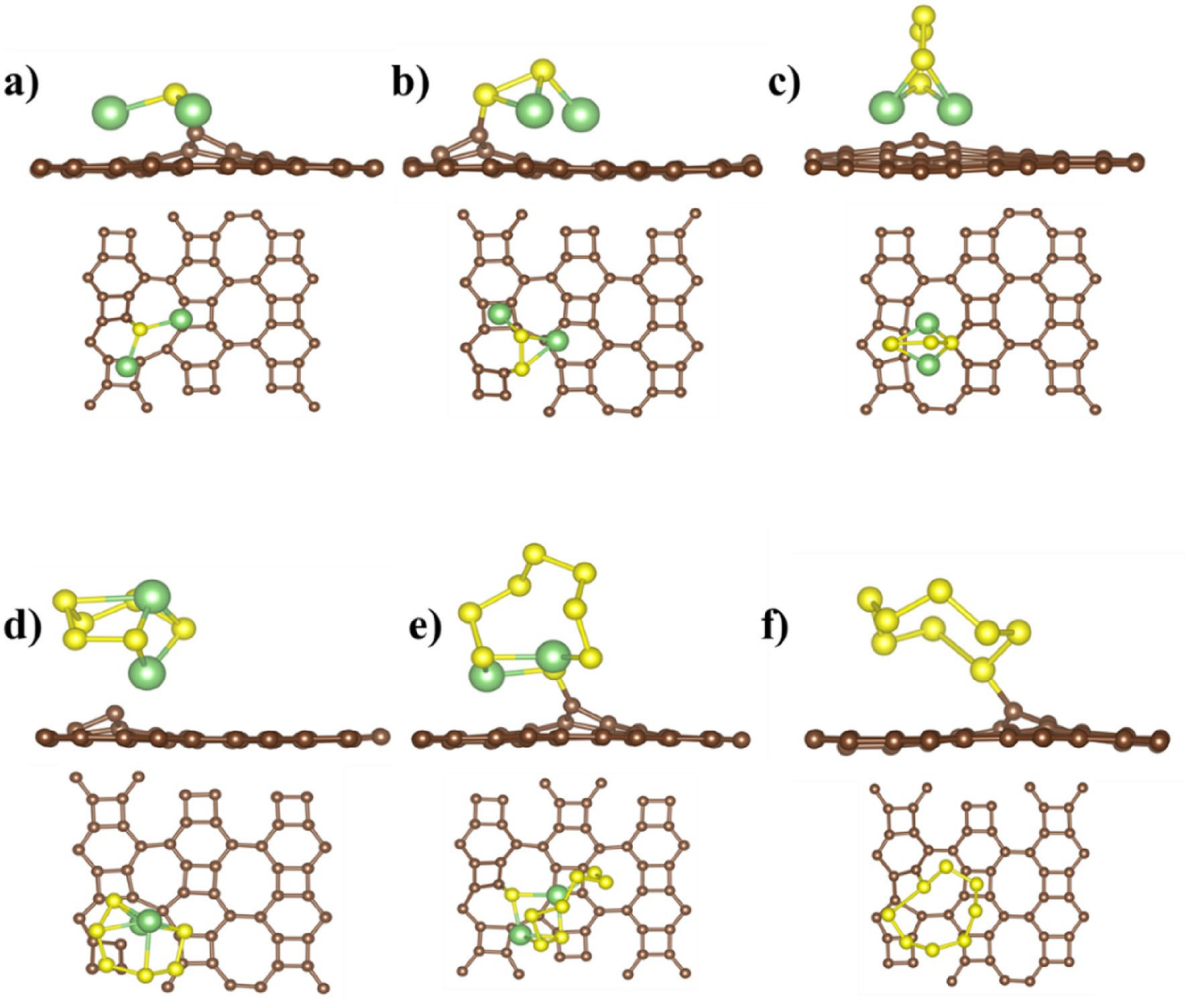
Ground state configuration of defective BPN sheet, (D1) adsorbed with (**a**) Li_2_S (**b**) Li_2_S_2_ (**c**) Li_2_S_4_ (**d**) Li_2_S_6_ (**e**) Li_2_S_8_ and (**f**) S_8_ clusters. The brown, green, and yellow spheres correspond to C, Li, and S atoms, respectively.

**Figure 4:**
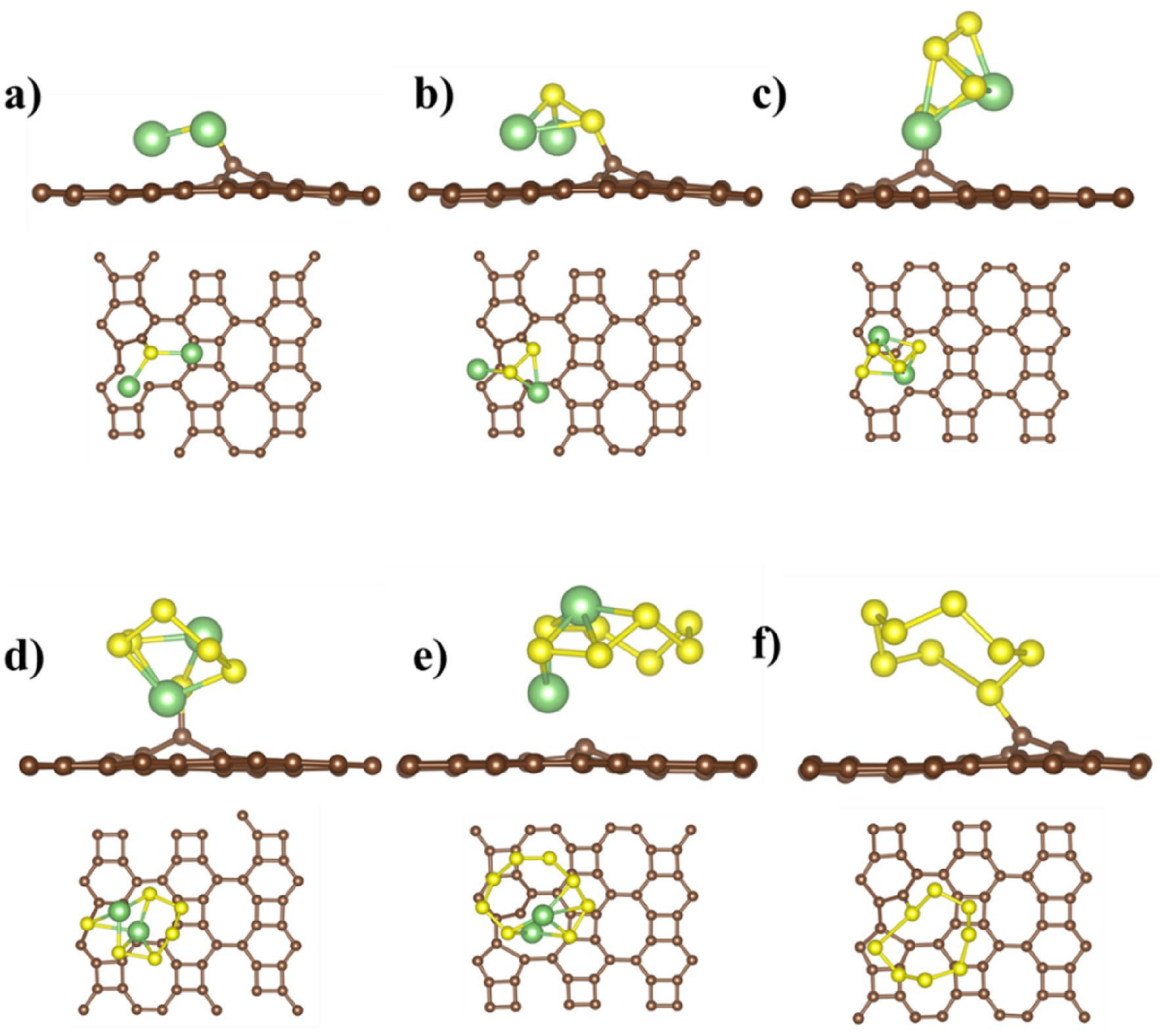
Ground state configuration of defective BPN sheet (D2) adsorbed with (**a**) Li_2_S (**b**) Li_2_S_2_ (**c**) Li_2_S_4_ (**d**) Li_2_S_6_ (**e**) Li_2_S_8_ and (**f**) S_8_ clusters. The brown, green, and yellow spheres correspond to C, Li, and S atoms, respectively.

The binding energy decreases gradually with the increase of the S atoms, but it also differs with the orientations of the molecule. When the S atom increased to 2 in Li_2_S_2_, the binding energy decreased by 0.342 eV because of the conformational changes noticed in the relaxed configurations. Two lithium atoms of Li_2_S are facing toward the sheet compared to only one lithium atom in Li_2_S_2_, directly resulting in lower binding energy. The highest binding energy values for each cluster with the corresponding favorable adsorption site are listed in Table 1, and the binding energy for each site is presented in the SI (see Fig S4), which ensures the stability of LiPSs on the BPN sheet. However, the binding energy values for the *x* > 3 are low and need further improvement. The binding energy of polysulfides is enhanced with defected BPN sheet. All the polysulfide clusters are placed at the top of the defect, and then the structures are relaxed, as shown in Figs. 3 and 4. The single C vacancy shows high charge localization (Fig. 1), shared with the Li_2_S_x_ and S_8_ clusters, resulting in higher binding energy than the pristine BPN sheet. For all Li_2_S_x_ clusters except with Li_2_S_6_ in defected BPN sheet, two Li atoms lie nearest to the surface. For Li_2_S_6,_ only one Li atom faces the surface, leading to the decreased binding energy. The S_8_ cluster is positioned at 1.73 Å from both defective surfaces, compared with a 3.49 Å in the pristine surface results increased binding energy of S_8_ from −0.21 eV (pristine BPN sheet) to −1.59 eV in the defective case. The minimum distances between the LiPSs clusters and the defective BPN sheets range from 1.67 Å (Li_2_S_8_) to 2.18 Å (Li_2_S_6_).

**Table 1:** Binding energies (in eV) of LiPSs and S_8_ clusters with pristine BPN (favorable adsorption site), defective BPN sheet, DOL, DME, and graphene.

	Pristine	D1	D2	DOL^31^	DME^32^	Graphene^33^
Li_2_S	−1.22 (S6)	−4.11	−3.60	–	–	−0.91
Li_2_S_2_	−0.89 (S6)	−2.92	−2.77	–	–	−0.74
Li_2_S_4_	−0.43 (S4)	−1.17	−1.52	−0.87	−0.92	−0.57
Li_2_S_6_	−0.61 (S6)	−1.07	−1.40	−0.90	−0.95	−0.53
Li_2_S_8_	−0.68 (S6)	−2.29	−0.72	−0.92	−0.92	−0.48
S_8_	−0.47 (S1)	−1.59	−1.28	–	–	−0.69

The leading cause of the shuttle effect in Li–S batteries is the smaller binding energy of LiPSs and S_8_ clusters with the cathode surface than with the electrolyte. Therefore, dissolution in the electrolytes and diffusion to the anode of LiPSs is easy. The binding energies of LiPSs with (1,3-dioxolane (DOL) and 1,2-dimethoxyethane (DME)) electrolytes are calculated. The binding energies of LiPSs with graphene are also listed to gain a more profound sense of BPN sheet as an anchoring material. The binding energies of LiPSs with DME and DOL molecules are smaller than those with the defected BPN sheet and higher than those with graphene, which indicates that graphene could not effectively trap LiPSs, but the defected BPN sheet can effectively trap LiPSs. Compared with other studied anchoring materials such as MXene (−0.83 eV to −4.31 eV)^17^, phosphorene (−0.75 eV to −2.51 eV)^29^, porous carbon allotropes (T-graphene (T-G) (−1.37 eV to −2.71 eV), Popgraphene (P-G) (−1.18 eV to e −1.91 eV ), Dodecagraphene (D-G) (−0.525 to −1.334), R-graphyne (R-G) (−0.735 to −1.009)^30^), the binding energy of LiPSs with the defected BPN sheet is more substantial, which indicates that the BPN sheet is indeed a promising anchoring material.

**Figure 5:**
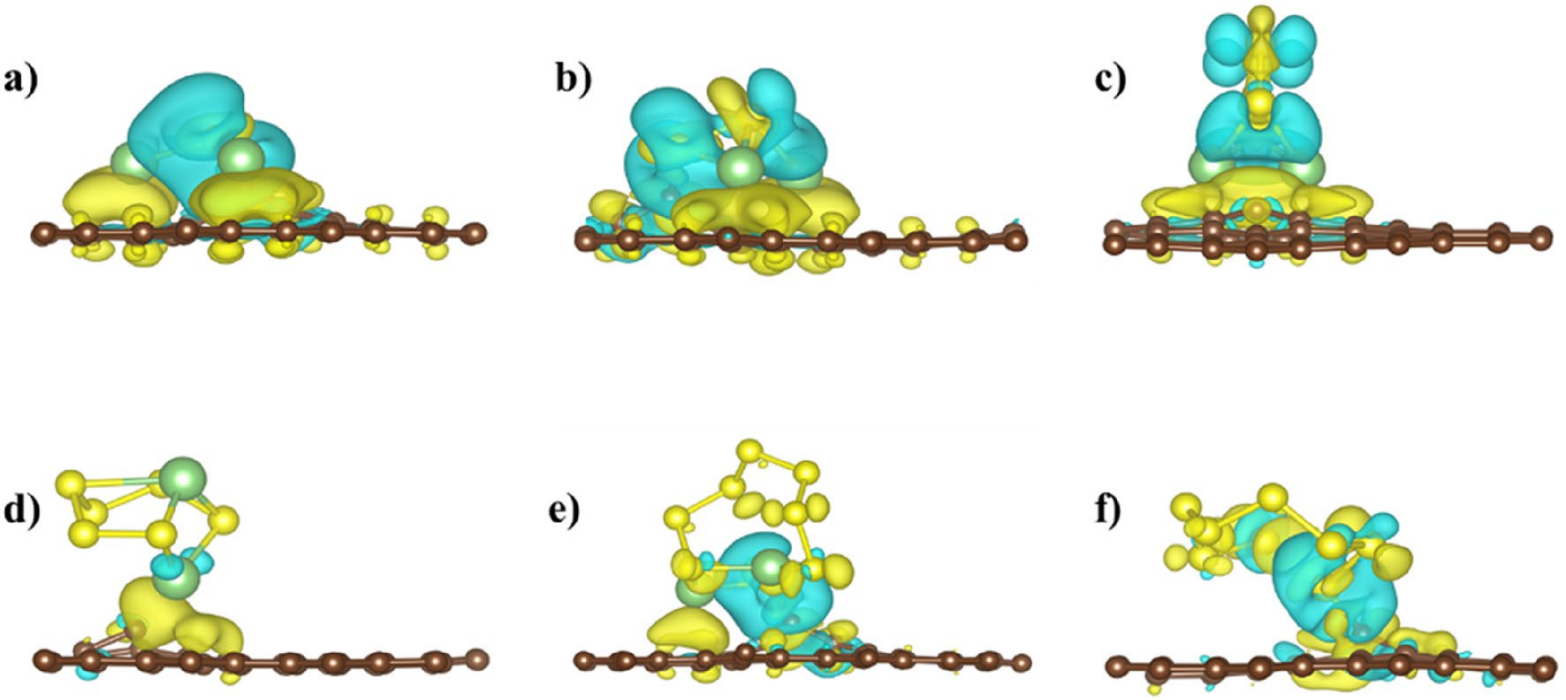
Calculated charge density difference of defective BPN sheet (D1 site) adsorbed with (**a**) Li_2_S, (**b**) Li_2_S_2_, (**c**) Li_2_S_4_, (**d**) Li_2_S_6_, (**e**) Li_2_S_8_, and (**f**) S_8_. Yellow and blue regions indicate charge accumulation and charge depletion, respectively, the iso-surface is set to 0.002 e/A^3^. The brown, green, and yellow spheres correspond to C, Li, and S atoms, respectively.

**Figure 6:**
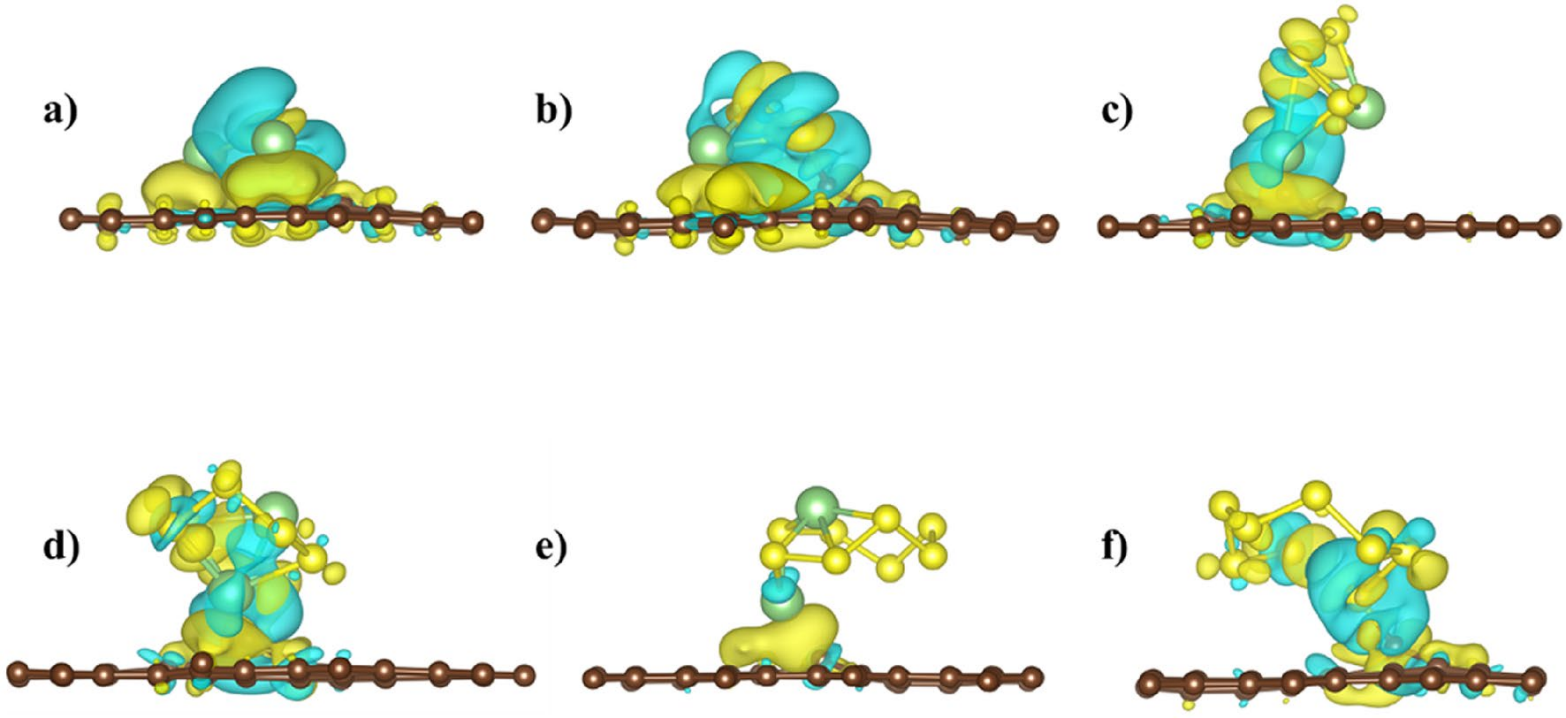
Calculated charge density difference of defective BPN sheet (D2 site) adsorbed with (**a**) Li_2_S, (**b**) Li_2_S_2_, (**c**) Li_2_S_4_, (**d**) Li_2_S_6_, (**e**) Li_2_S_8_, and (**f**) S_8_. Yellow and blue regions indicate charge accumulation and depletion, respectively, the iso-surface value is 0.002 e/A^3^. The brown, green, and yellow spheres correspond to C, Li, and S atoms, respectively.

Defected BPN sheet outperforms the pristine sheet in the binding energy of LiPSs and S_8_ clusters. The Bader analysis shows that the LiPSs and S_8_ donate the charge of between −0.05 *e* to −1.02 *e* to the BPN sheet (see Table 2). The charge is mainly accumulated (depleted) on S (Li) before absorption and transferred substantially to the sheet after absorption of clusters. The charge density difference between LiPSs and the defective BPN sheet is calculated according to $\Delta \rho ={\rho }_{total}-{\rho }_{defectiveBPN}-{\rho }_{LiPSs/S_{8}}$, where ${\rho }_{total}$, ${\rho }_{defectiveBPN}$, and ${\rho }_{LiPSs/S_{8}}$ are the calculated charge density of combined BPN sheet and LiPSs/S_8_ systems, defected BPN sheet alone, and the isolated LiPSs/S_8_ clusters, respectively. The calculated charge density difference of the defective BPN sheet is shown in Figs. 5 and 6.

**Table 2:** Calculated charge transferred (in e) to the defected BPN sheet from LiPSs and S_8_ cluster after adsorption.

	Li_2_S	Li_2_S_2_	Li_2_S_4_	Li_2_S_6_	Li_2_S_8_	S_8_
D1	−1.13	−1.06	−0.74	−0.05	−0.07	−0.05
D2	−1.02	−1.01	−0.21	−0.08	−0.14	−0.05

Figure 5 shows, Li_2_S_6_ has the least apparent charge redistribution compared to the other clusters. For Li_2_S_x_ (*x* = 1, 2, 4, 8) and S_8_ adsorption, electrons are mainly accumulated in the region between Li and S nearest. The detailed charge rearrangement on the constituents of LiPSs/S_8_ Species before and after adsorption on BPN sheet are listed in the SI (Table S1). Li_2_S positioned itself at 1.71 Å from the defective BPN sheet in the D1 case, where two lithium atoms facing the surface led to the highest charge transferred value. Moreover, most carbon materials have low dipole moments, while Li_2_S and Li_2_S_2_ have high dipole moments. When the BNP sheet interacts with Li_2_S and Li_2_S_2_, a strong dipole–dipole electrostatic interaction results in the highest charge transfer between them^34,35^. On the contemporary, Li_2_S_6_ has the lowest charge transferred within the lithium polysulfides and its highest distance, 2.18 Å, on top of the D1 defective BPN sheet.

## Conclusion

In summary, the density functional theory calculations are carried out to explore the potential and performance of 2D BPN sheet as an anchoring material for Li–S batteries. The results show that the defective BPN sheet significantly improves the binding of LiPSs with binding energies ranging from −1.07 to −4.11 eV, which can effectively inhibit the shuttle effect and reduce the migration barrier to realize the rapid realization charge/discharge. The Bader charge analysis shows an electronic charge transfer ranging from −0.05 e to −1.12 e from LiPSs to the BPN sheet. Compared to graphene, MXenes, and other potential anchoring materials, the BPN sheet has the highest binding energies, making it a more efficient and reliable choice. Our work deepens the fundamental understanding of the anchoring mechanism and demonstrate that the biphenylene sheet has excellent potential to be an outstanding anchoring material for Li–S batteries for suppressing the shuttle effect.

## Supplementary Information

Supplementary Information.

## Data Availability

No datasets were generated or analysed during the current study.
