# Supplementary material for: Two-dimensional biphenylene: a promising anchoring material for lithium-sulfur batteries
Source: Sci Rep. 2022 Mar 17;12:4653. doi: 10.1038/s41598-022-08478-5 (PMC8931010; doi:10.1038/s41598-022-08478-5)
Supplement: Supplementary file 1 — Supplementary Information. [file 41598_2022_8478_MOESM1_ESM.docx]

**Supporting Information**

**Two-dimensional Biphenylene: A Promising Anchoring Material for Lithium-Sulfur Batteries**

Hiba Khaled Al-Jayyousi^a^, Muhammad Sajjad^b^, Kin Liao^c^, Nirpendra Singh^b*^

*^a^Department of Mechanical Engineering, Khalifa University of Science and* *Technology, Abu Dhabi-127788, United Arab Emirates (UAE)*

*^b^Department of Physics, Khalifa University of Science and Technology, Abu Dhabi-127788, United Arab Emirates (UAE)*

*^c^Department of Aerospace Engineering, Khalifa University of Science and Technology, Abu Dhabi-127788, United Arab Emirates (UAE)*


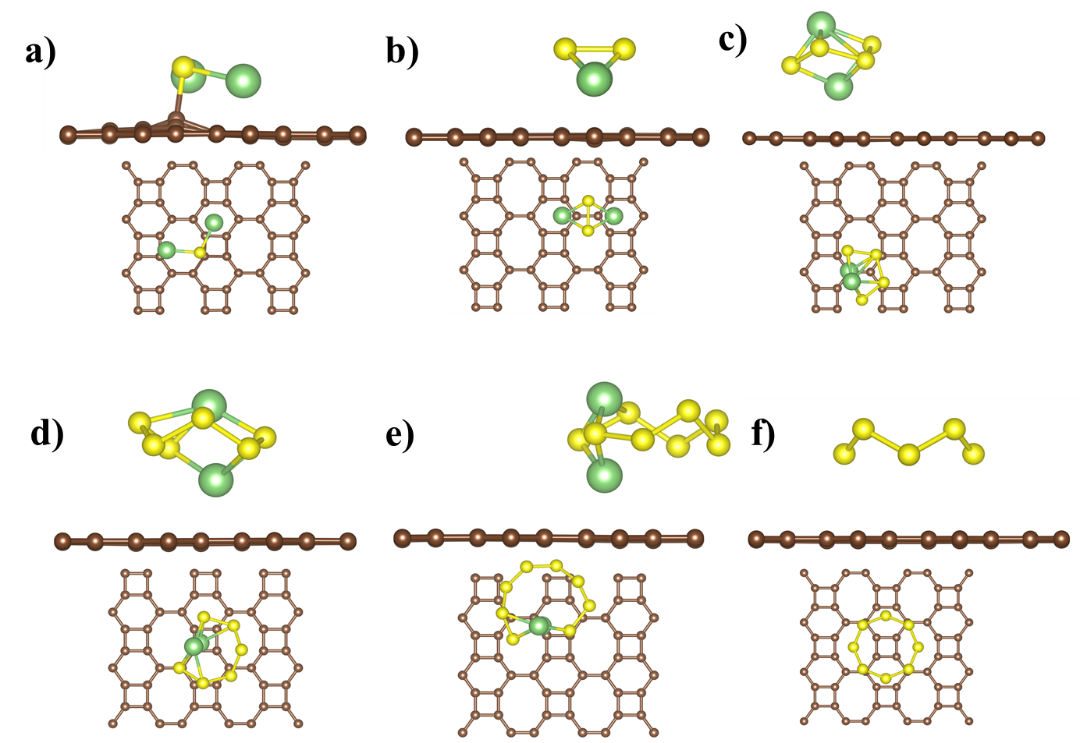


Figure S1: The most stable configuration of the BPN sheet adsorbed with (a) Li_2_S, (b) Li_2_S_2_, (c) Li_2_S_4_, (d) Li_2_S_6_, (e) Li_2_S_8_, and (f) S_8_. Brown, green, and yellow spheres represent C, Li, and S atoms, respectively.

Figure S2: The calculated density of states of 3 × 3 × 1 (a) pristine BPN sheet (b) BPN sheet with D1 defect, and (c) BPN sheet with D2 defect.


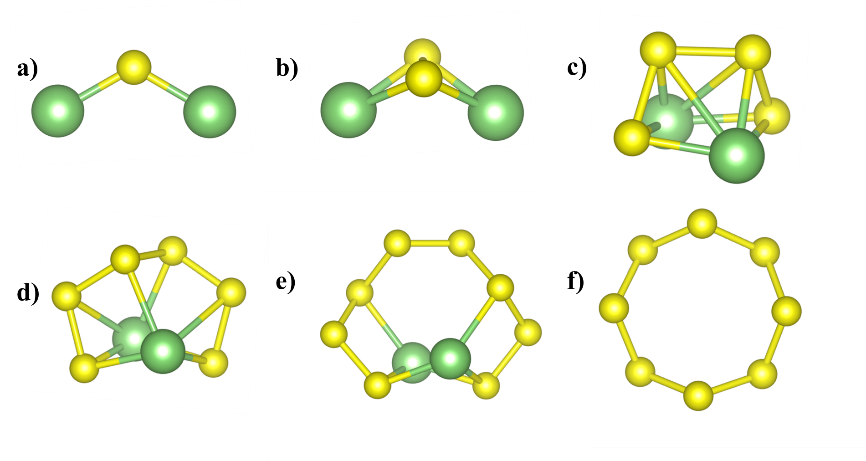


Figure S3: The ground state structure of (a) Li2S (b) Li2S2 (c) Li2S4 (d) Li2S6 (e) Li2S8 and (f) S8 clusters. The green, and yellow spheres correspond to Li and S atoms, respectively.

**
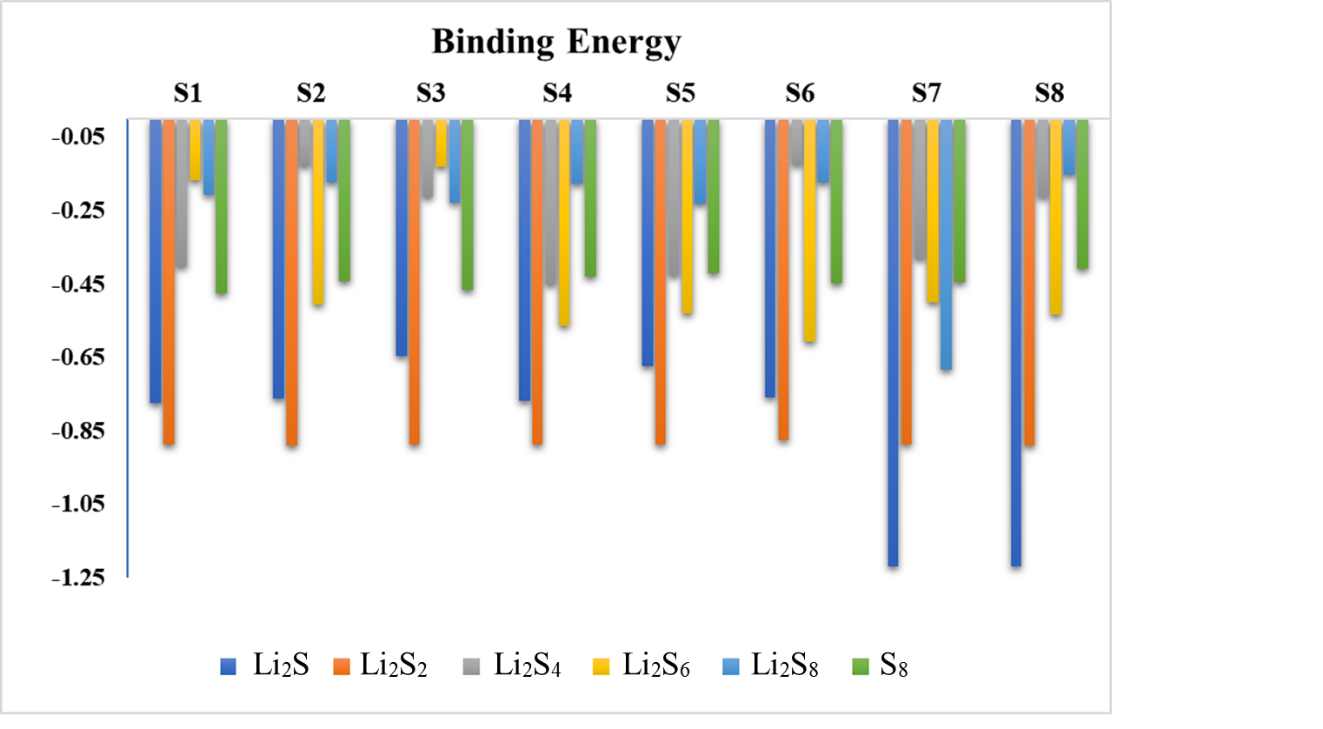
**

Figure S4: Calculated binding energy of Li_2_S_x_ (x=1, 2, 4, 6, 8) and S_8_ on eight different adsorption sites of the BPN sheet.

| *Table S1: Charge Rearrangement (in e) on the constituents of LiPSs/S_8_ Species after (before) adsorption on BPN sheet.* | | | | | | | | |
| --- | --- | --- | --- | --- | --- | --- | --- | --- |
| **D 1** | | | | | **D2** | | | |
|  | **∑S** | **Li1** | **Li2** | **∑** | **∑S** | **Li1** | **Li2** | **∑** |
| **Li_2_S** | -0.46  ( -1.72) | 0.79 (0.86) | 0.80 (0.86) | -1.13 | -0.48 | 0.72 | 0.78 | -1.02 |
| **Li_2_S_2_** | -0.42  (-1.73) | 0.71 (0.87) | 0.78 (0.87) | -1.06 | -0.37 | 0.77 | 0.63 | -1.01 |
| **Li_2_S_4_** | -0.73  ( -1.72) | 0.70  (0.86) | 0.78 (0.86) | -0.74 | -0.71 | 0.41 | 0.51 | -0.21 |
| **Li_2_S_6_** | -1.12  (-1.75) | 0.50 (0.87) | 0.68 (0.87) | -0.05 | -0.84 | 0.46 | 0.46 | -0.08 |
| **Li_2_S_8_** | -0.74  (-1.74) | 0.32  (0.87) | 0.49 (0.87) | -0.07 | -1.80 | 0.97 | 0.98 | -0.14 |
